# Pattern of omega-3 polyunsaturated fatty acid intake and fish consumption and retinal vascular caliber in children and adolescents: A cohort study

**DOI:** 10.1371/journal.pone.0172109

**Published:** 2017-02-13

**Authors:** Bamini Gopinath, Hanieh Moshtaghian, Victoria M. Flood, Jimmy C. Y. Louie, Gerald Liew, George Burlutsky, Paul Mitchell

**Affiliations:** 1 Center for Vision Research, Department of Ophthalmology and The Westmead Institute, University of Sydney, Sydney, NSW, Australia; 2 School of Medicine, Faculty of Science, Medicine and Health, University of Wollongong, Wollongong, NSW, Australia; 3 St Vincent’s Hospital, Sydney, NSW, Australia; 4 Faculty of Health Sciences, University of Sydney, Sydney, NSW, Australia; 5 School of Biological Sciences, Faculty of Science, The University of Hong Kong, Hong Kong; 6 School of Life and Environmental Sciences, University of Sydney, Sydney, NSW, Australia; The Pennsylvania State University, UNITED STATES

## Abstract

We aimed to investigate whether fish and long chain omega-3 polyunsaturated fatty acid (LCn-3 PUFA) consumption changed appreciably during adolescence. We also assessed whether these dietary variables are associated with retinal microvascular signs (possible markers of future cardiovascular disease risk). 633 children had dietary data at ages 12 and 17. Fish and LCn-3 PUFA [eicosapentaenoic acid (EPA), docosapentaenoic acid (DPA) and docosahexaenoic acid (DHA)] intake was assessed by a food frequency questionnaire. Retinal vessel caliber was quantified from digital photographs using computer software. Mean energy-adjusted intakes (mg/day) of total LCn-3 PUFA, EPA, and DHA at age 12 were 297.1±261.1; 102.5±106.9; and 129.7±137.7, respectively; and this increased significantly at age 17 to: 347.0±324.0 (p<0.0001); 122.5±132.7 (p = 0.0001); and 160.3±171.4 (p <0.0001), respectively. Increasing quartiles of LCn-3PUFA intake were associated with widening of mean retinal arteriolar caliber among 17-year old girls ~3.9 μm (multivariable-adjusted *P*-trend = 0.001). Girls who consumed ≥2 serves of fish/week versus those who did not had ~2.1 μm wider retinal arterioles (p = 0.03). No associations were observed among boys or with retinal venules. Mean dietary intakes of LCn-3 PUFA increased during adolescence, but are still below recommended levels of consumption. These results suggest that LCn-3 PUFA and fish intake might have a beneficial influence.

## Introduction

Long chain omega-3 polyunsaturated fatty acids (LCn-3 PUFA) have been associated with several health benefits during various stages of human life including neurological function, psychological health [1,2], autoimmune and inflammatory disease including cardiovascular complications [3–5] which can benefit from LCn-3 PUFA intake. Fish and seafood products are one of the main sources of LCn-3 PUFA [eicosapentaenoic acid (EPA), docosapentaenoic acid (DPA) and docosahexaenoic acid (DHA)] [6]. Therefore, fish and seafood consumption has also been shown to have beneficial effects on health including reduced risk of stroke, atrial fibrillation and coronary heart disease [7–9].

Despite known health benefits of LCn-3 PUFA and fish intake, children and adolescents in most countries such as the UK [10] and Australia [11,12] have low LCn-3 PUFA intake. Data from 1995 National Nutrition Survey (NNS) [13], and the 2007 Australian National Children’s Nutrition and Physical Activity Survey (ANCNPAS) [14] suggest that Australian children and adolescents have low fish and LCn-3 PUFA consumption. However, these studies did not consider follow-up of the same group of children to investigate whether there is a change in LCn-3 PUFA intake and fish consumption during childhood and adolescence. This information is important in trying to develop effective public health and nutrition programmes that aim to improve LCn-3 PUFA intake and fish consumption levels among schoolchildren.

Moreover, although evidence suggest that diets rich in LCn-3 PUFA and fish could have advantageous effects on both microvasculature and microvasculature in adults [7–9,15]; there is limited data on these relationships in children and adolescents. The key retinal microvascular changes associated with cardiovascular disease (CVD) in adults are narrower retinal arteriolar caliber and wider venular calibre [16–18]. These structural changes are also associated with CVD risk factors, including obesity and elevated blood pressure (BP) [19–21]. In adults, we have shown that regular fish in the diet (eaten at least twice per week) was associated with slight widening of mean retinal arteriolar diameter and slight narrowing of mean retinal venular diameter [15]. Recently, a study of children and adolescents with type 1 diabetes, showed that a dietary pattern characterized by lower intake of vegetables and fish was associated with wider retinal venular calibre in children and adolescents with type 1 diabetes [22]. Further research is warranted to investigate whether LCn-3 PUFA and fish consumption independently influence the retinal microvasculature in young, healthy children who are largely free of known systemic cardiovascular diseases. These epidemiological data are potentially important because subtle retinal microvascular signs may be a sign of microcirculatory health [18], and the presence of this risk factor in childhood and/or adolescence could contribute to future development of targeted interventions

Therefore, this analysis of a cohort of Australian schoolchildren aged 12 and 17 aimed to address these gaps in knowledge by examining the: 1) pattern and change in LCn-3 PUFA intake (EPA, DPA and DHA) and fish/ seafood consumption during adolescence from age 12 to 17; and 2) cross-sectional associations of LCn-3 PUFA intake and fish/ seafood consumption with retinal microvascular calibre at age 12 and 17.

## Methods

### Study population

The Sydney Childhood Eye Study is a population-based survey of eye conditions and other health outcomes in schoolchildren living within the Sydney Metropolitan Area, Australia. It was approved by the Human Research Ethics Committee, University of Sydney, the Department of Education and Training, and the Catholic Education Office, New South Wales, Australia [23]. All study methods were carried out in accordance with these approved guidelines. We obtained informed written consent from at least one parent of each child, as well as the verbal assent and written consent from each child/ adolescent before the examinations. Study methods have been previously described [23]. Briefly, students with a mean age 12.7 years in a stratified random cluster sample of 21 high schools across Sydney were eligible to participate. Stratification was based on socioeconomic status data and led to a proportional mix of public, private or religious high-schools. Of the 3144 eligible 12-year-old children, 2367 were given parental permission to participate and 2353 underwent examinations (74.9%). Data for the 12-year-old cohort were collected during 2004–5 and then 5 years later during 2009–11; 1216 were re-examined (51.7% of baseline participants).

### Dietary data assessment

Dietary data were collected using a 120-item self-administered, semi-quantitative food-frequency questionnaire, designed for specific use in Australian children and adolescents [24], administered at ages 12 and 17. The validity of this food-frequency questionnaire has previously been reported [24]. For instance, the de-attenuated, energy adjusted Pearson’s correlation coefficient for dietary fats was 0.53, and the proportion of individuals correctly classified within one quintile for dietary fats was 59%, when compared to weighed food records [24]. The median value (50^th^ percentile cut point) for PUFA intake from the FFQ and food records were similar– 8 and 9, respectively. The 25^th^ percentile cut-points for PUFA intake from the FFQ and food records were also similar– 5 and 8, respectively. While the 75^th^ percentile cut-points for PUFA intake from the FFQ and food records was the same i.e. 11. In terms of total fat intake similar median values were also observed between the FFQ and food records, 86 and 83, respectively. The 25^th^ percentile cut-points for total fat intake from the FFQ was slightly lower than from the foods records i.e. 57 and 71, respectively. While the 75^th^ percentile cut-points (upper IQR) for total fat intake from the FFQ and food records were comparable—110 and 98, respectively [24].

Food-frequency questionnaire items were translated into daily food and nutrient intakes using a purpose-built query in Microsoft Access 2007, using various nutrient databases [25–27]. Subjects who reported an energy intake <2,090 kJ/ day or >20,900 kJ/ day (n = 71) were excluded from the analysis, consistent with previous studies [24]. Further, we inspected extreme nutrient values (upper and lower 1% in distribution) among those with plausible energy intake to correct any data entry errors and to check for plausibility. Missing values for LCn-3PUFAs (EPA, DPA and EPA) were also replaced with AUSNUT2007 [27] or Royal Melbourne Institute of Technology fatty acid database data [28]. Furthermore, data for some items were created by analysing recipes in FoodWorks, version 7, 2012 software (Xyris software, Brisbane, Australia). For the current study we are only able to determine LCn-3PUFA from diet only, and the FFQ we administered did not take into account omega-3 supplement usage. We determined the number of study participants meeting the Australian National Heart Foundation (ANHF) recommendation of two or more serves of oily fish per week and total intake EPA + DHA intake of 500 mg/day or more [29] at age 17.

### Retinal photography and analysis

Participants had four dilated, digital photographs taken including the optic disc and macula of each eye using a Canon 60UVID10 fundus camera (Canon Inc., Tokyo, Japan). Retinal vascular caliber measurements for the right eye of each child were used. Left eye measurements were used if the photographs of the right eye were un-gradable. One grader, masked to participant identity and characteristics, measured retinal vessel caliber using a computer-assisted program with high reproducibility, this has been previously described [30,31]. Average retinal arteriolar and venular calibers were calculated using the Knudtson–Hubbard formula [32]. We were unable to prospectively analyze retinal vessels, as cameras with slight differences in magnification were used to obtain digital retinal photographs at baseline (age 12) and 5 years later at follow-up (age 17). There is currently no correction factor available that could be applied to allow digital photos taken at both examinations to be compared; hence, longitudinal comparison of retinal vessel measures is not valid in this instance and only cross-sectional analysis are presented.

### Assessment of confounders

Information on all covariates was obtained both at baseline (age 12) and at the 5-year follow-up (age 17). Parents also completed a comprehensive 193-item questionnaire. Socio-demographic information covering ethnicity of the child, country of birth, education, occupation and parental age of both parents was collected. The ethnicity of the child was determined only if both parents shared that ethnic origin. Otherwise, children were placed in a mixed ethnicity category. Ethnicity was classified on the basis of self-identification by the parents, combined with information about the place of birth of the child [33].

Each participant’s height was measured to the nearest 0.1 cm with shoes off using a freestanding SECA height rod (Model 220, Hamburg, Germany). Weight in kilograms was measured to the nearest 0.1 kg using a standard portable weighing machine, after removing any heavy clothing. Anthropometric measures were recorded up to 2 decimal points. BP was measured on the school premises according to a standard protocol [23]. After 5-min resting, BP was measured in a seated position using an automated sphygmomanometer (HEM 907; Omron Healthcare Inc., Tokyo, Japan) with appropriate cuff size. We followed general recommendations on selecting cuff size to ensure that the bladder length was approximately 80% and width was at least 40% of the arm circumference, covering the upper arm without obscuring the antecubital fossa [34]. Three separate BP measurements were taken (with a resting time of 5-min between each measurement), and averaged for analysis [35]. Mean arterial BP (MABP) was calculated as one-third of the systolic plus two thirds of the diastolic BP. Axial length of the eye was measured before cycloplegia using an optical biometer (IOLMaster; Carl Zeiss Meditec, Oberkochen, Germany), using dual-beam partial coherence interferometry.

### Statistical analyses

Statistical analyses were performed using SAS (v9.2, SAS Institute, NC). Descriptive analyses were used to describe participants’ characteristics and their fish and LCn-3PUFA intake at both baseline and follow up. Absolute energy-adjusted dietary intake of fish and seafood, LCn-3PUFA, EPA, DPA and DHA were compared between baseline and follow-up. Paired sample t-tests were used to assess changes in individual intakes over time. We used mixed models to adjust for cluster sampling effects in all analyses. Logistic regression analysis was used to assess longitudinal associations between various socioeconomic correlates (age, sex, ethnicity, parental education and employment status) at age 12 and dietary intakes of LCn-3PUFA and fish at age 17. All socioeconomic correlates were included in the one logistic regression model.

Cross-sectional linear regression analyses of retinal vessel caliber compared adjusted means across quartiles of LCn-3PUFA, EPA, DPA and DHA and fish intake. Adjusted mean retinal vessel caliber was also compared between groups who met the national dietary recommendations for EPA + DHA intake and fish consumption. Covariates that were found to be significantly associated with retinal vessel caliber [36] were included in multivariable linear regression models: age, sex, ethnicity, total energy intake, axial length of the eye, BMI, MABP and fellow vessel caliber (i.e. venular caliber adjusted in model for arteriolar caliber and vice versa).

Linear regression analyses indicated interactions between sex and the associations of fish consumption with retinal arteriolar caliber (*P*_interaction_ = 0.05). Analyses of retinal vessels were therefore stratified by sex.

## Results

### Pattern and change in LCn-3 PUFA intake and fish consumption

Of the 2353 schoolchildren who were examined at baseline, 633 participants were followed up 5 years later and had complete dietary and retinal measures. Baseline characteristics of study participants versus non-participants (n = 1720) are shown in the S1 Table. Participants versus non-participants were older, more likely to be Female, Caucasian or East Asian, have parents who had tertiary level education and in employment, and have lower BMI. Table 1 shows the study characteristics of 12- and 17-year old participants stratified by gender. 12-year old boys compared to girls were more likely to be older, Caucasian and have greater energy intake and axial length but less likely to be East Asian ethnicity. 17-year old boys compared to girls were more likely to be older, Caucasian and have greater energy intake, axial length and MABP, but less likely to be East Asian ethnicity (Table 1). A significant increase in mean energy-adjusted dietary intakes of LCn-3 PUFA, EPA and DHA was observed from age 12 to 17 years in the overall cohort (n = 633), and in boys and girls separately (Table 2). No significant change in mean DPA intake during adolescence was observed (Table 2). At age 12 the proportion of schoolchildren who consumed fish 1 or more times per week was 57.0% and this increased slightly to 59.9% by age 17, however, this was non-significant (p = 0.14). Fish consumption did not change appreciably in girls (56.7 versus 59.5 g/day; p = 0.23) or boys (57.5 versus 60.3 g/day; p = 38) over the 5 years.

**Table 1:** Baseline characteristics of 12- and 17-year old children (n = 633), stratified by gender.

Characteristics	12-year old			17-year old		
	Boys	Girls	P	Boys	Girls	P
Age	12.8 (0.4)	12.7 (0.4)	0.02	17.4 (0.5)	17.2 (0.4)	<0.0001
Ethnicity, %						
Caucasian	201 (71.3)	212 (60.4)	0.004	201 (71.3)	212 (60.4)	0.004
East Asian	34 (12.1)	70 (19.9)	0.01	34 (12.1)	70 (19.9)	0.01
South Asian	13 (4.6)	18 (5.1)	0.76	13 (4.6)	18 (5.1)	0.76
Middle Eastern	12 (4.3)	22 (6.3)	0.26	12 (4.3)	22 (6.3)	0.26
Other	22 (7.8)	29 (8.3)	0.26	22 (7.8)	29 (8.3)	0.26
Parental education^a^	132 (50.4)	154 (47.0)	0.41	127 (52.7)	151 (49.5)	0.46
Parental employment ^b^	249 (95.4)	298 (91.7)	0.07	232 (95.6)	288 (94.1)	0.36
BMI	19.9 (3.9)	20.2 (4.4)	0.27	22.6 (4.1)	22.4 (4.6)	0.60
MABP	80.8 (7.6)	81.5 (8.4)	0.27	89.1 (8.7)	84.4 (9.2)	<0.0001
Axial length	23.6 (0.8)	23.1 (0.9)	<0.0001	23.6 (0.8)	23.1 (0.9)	<0.0001
Energy intake	9330.9 (3642)	8304.9 (3121)	0.0002	9378.4 (3350)	8084.0 (3008)	<0.0001

Data is presented as mean (SD) or n (%).

^a^ Parents who had attained tertiary level education (greater than high school).

^b^ Parents who were in full or part-time employment.

**Table 2:** Energy-adjusted dietary intakes of long chain omega-3 polyunsaturated fatty acids (LCn-3 PUFA) at age 12 and 17 years among participants of the Sydney Childhood Eye Study during 2004–5 to 2009–11.

LCn-3 PUFA (mg/ day)	12 years	17 years	Change in intake	*p*-value
	Mean (±SD)	Mean (±SD)	Mean (±SE)	
All (n = 633)				
Total LCn-3PUFA	297.1 (261.1)	347.0 (324.0)	49. (12.5)	<0.0001
EPA	102.5 (106.9)	122.5 (132.7)	19.9 (5.1)	0.0001
DPA	63.4 (28.2)	63.2 (28.6)	-0.2 (1.2)	0.88
DHA	129.7 (137.7)	160.3 (171.4)	30.7 (6.6)	<0.0001
Girls (n = 351)				
Total LCn-3PUFA	309.0 (259.2)	357.6 (318.1)	48.6 (16.2)	0.003
EPA	107.8 (105.6)	127.1 (129.7)	19.3 (6.5)	0.004
DPA	65.2 (28.5)	64.0 (28.3)	-1.1 (1.7)	0.49
DHA	134.5 (137.0)	165.5 (168.2)	31.0 (8.5)	0.0003
Boys (n = 282)				
Total LCn-3PUFA	282.4 (263.2)	333.9 (331.2)	57.4 (21.7)	0.01
EPA	96.0 (108.2)	116.7 (136.3)	20.7 (8.1)	0.01
DPA	61.2 (27.6)	62.2 (29.0)	1.0 (1.8)	0.56
DHA	123.7 (138.5)	153.9 (175.3)	30.3 (10.3)	0.003

Baseline sociodemographic predictors of meeting the national recommendation of two or more serves of oily fish per week and total intake EPA + DHA intake of 500 mg/day or more [29] at age 17 was examined. East Asian children compared to Caucasian were 87% more likely to meet the recommendations for EPA + DHA intake, OR 1.87 (95% CI 1.00–3.47). East Asian children and children from ‘other’ ethnicities compared to Caucasian children also had greater odds of eating the recommended levels of fish at age 17, OR 3.50 (95% CI 2.16–5.68) and OR 1.96 (95% CI 1.02–3.76), respectively. We determined the interaction between gender and ethnicity on meeting the national recommendation of two or more serves of oily fish per week, however, the interaction was not significant (*P*_interaction_ = 0.54). This suggests that the influence of gender and ethnicity did not depart from the multiplicative scale of the influence of each factor alone on fish consumption. The other sociodemographic factors such as age, gender, parental education and employment status were not significantly associated with meeting the recommendations for fish or EPA + DHA intake during adolescence (data not shown).

### Cross-sectional associations of LCn-3 PUFA intake and fish/ seafood consumption with retinal vascular calibre

At age 12, we found no significant cross-sectional associations between consuming the recommended amount of EPA + DHA and fish and retinal vessel caliber (Table 3). However, Table 4 shows that 17-year old girls who met the recommended intake of EPA and DHA compared to those who did not meet this recommendation had ~2.5 μm wider retinal arterioles (p = 0.04). Similarly, girls who consumed 2 or more serves of fish per week versus those who did not had ~2.1 μm wider retinal arterioles (p = 0.03). There was a marginally significant association between meeting the recommendations for fish consumption and narrower retinal venules (~2.6 μm) in girls (p = 0.07). Significant associations were not observed in 17-year old boys (Table 4). Table 5 shows that with increasing dietary intake of LCn-3PUFA, EPA and DHA (i.e. from quartile 1 to 4), there was significant increase in retinal arteriolar caliber among girls: ~3.9 μm (*P*-trend = 0.001); ~3.7 μm (*P*-trend = 0.0004); and ~3.3 μm (*P*-trend = 0.003), respectively. There was a marginally significant association between increasing intake of dietary EPA (from the 1^st^ to the 4^th^ quartile of intake) and widening of retinal venular caliber in girls 2.9 μm (*P*-trend = 0.05). No significant associations were observed in boys (Table 6).

**Table 3:** Cross-sectional association between recommended intakes of fish consumption and EPA and DHA intakes^a^ and adjusted mean retinal vascular caliber in 12 year old participants.

	Retinal vascular caliber, mean (95% CI) ^b^	
Dietary intake	Arteriolar caliber, μm	Venular caliber, μm
**All (n = 1920)**		
EPA + DHA ≥500 mg/day		
No (n = 1741)	151.1 (150.1–152.2)	219.6 (218.2–221.1)
Yes (n = 179)	151.2 (149.4–153.0)	220.5 (217.9–223.1)
*p*-value	0.91	0.48
Fish intake ≥2 serves/week		
No (n = 1418)	151.2 (150.1–152.4)	219.6 (218.0–221.1)
Yes (n = 502)	150.8 (149.5–152.2)	220.1 (218.3–222.0)
*p*-value	0.51	0.51
**Girls (n = 975)**		
EPA + DHA ≥500 mg/day		
No (n = 895)	152.7 (151.5–153.9)	221.3 (219.6–223.0)
Yes (n = 80)	153.6 (151.0–156.3)	223.8 (220.0–227.5)
*p*-value	0.47	0.19
Fish intake ≥2 serves/week		
No (n = 731)	152.8 (151.5–154.0)	221.6 (219.7–223.4)
Yes (n = 244)	152.8 (151.1–154.4)	221.5 (219.1–223.9)
*p*-value	1.00	0.96
**Boys (n = 945)**		
EPA + DHA ≥500 mg/day		
No (n = 846)	149.8 (148.5–151.2)	217.5 (215.5–219.6)
Yes (n = 99)	149.0 (146.7–151.4)	217.4 (214.1–220.8)
*p*-value	0.49	0.95
Fish intake ≥2 serves/week		
No (n = 687)	149.9 (148.5–151.4)	217.2 (215.1–219.3)
Yes (n = 258)	149.2 (147.4–150.9)	218.3 (215.8–220.8)
*p*-value	0.35	0.32

^a^Australian National Heart Foundation (ANHF) recommendation of two or more serves of oily fish per week and EPA and DHA intake of 500 mg/day or more [29].

^b^ Adjusted for age, sex, ethnicity, energy intake, body mass index, mean arterial blood pressure, axial length, and fellow vessel caliber. Sex was not adjusted in the final model when analyzing boys and girls separately.

**Table 4:** Cross-sectional association between recommended intakes of fish consumption and EPA and DHA intakes^a^ and adjusted mean retinal vascular caliber in 17-year old participants.

	Retinal vascular caliber, mean (95% CI) ^b^	
Dietary intake	Arteriolar caliber, μm	Venular caliber, μm
**All (n = 1199)**		
EPA + DHA ≥500 mg/day		
No (n = 1052)	160.7 (160.1–161.3)	233.0 (231.6–234.3)
Yes (n = 147)	162.2 (160.6–163.9)	232.0 (229.3–234.8)
*p*-value	0.09	0.50
Fish intake ≥2 serves/week		
No (n = 856)	160.6 (159.9–161.3)	232.9 (231.5–234.3)
Yes (n = 343)	161.5 (160.4–162.6)	232.8 (230.8–234.8)
*p*-value	0.20	0.93
**Girls (n = 663)**		
EPA + DHA ≥500 mg/day		
No (n = 575)	162.3 (161.0–163.6)	237.8 (235.6–240.1)
Yes (n = 88)	164.8 (162.4–167.1)	235.9 (232.1–239.7)
*p*-value	0.04	0.29
Fish intake ≥2 serves/week		
No (n = 475)	162.0 (160.6–163.4)	238.3 (236.0–240.7)
Yes (n = 188)	164.1 (162.3–165.9)	235.7 (232.8–238.6)
*p*-value	0.03	0.07
**Boys (n = 536)**		
EPA + DHA ≥500 mg/day		
No (n = 477)	158.8 (157.3–160.1)	234.5 (232.1–236.8)
Yes (n = 59)	159.1 (156.5–161.7)	233.5 (229.5–237.6)
*p*-value	0.80	0.64
Fish intake ≥2 serves/week		
No (n = 381)	159.0 (157.5–160.5)	234.5 (232.0–236.9)
Yes (n = 155)	158.4 (156.6–160.2)	234.1 (231.3–237.0)
*p*-value	0.51	0.82

^a^Australian National Heart Foundation (ANHF) recommendation of two or more serves of oily fish per week and EPA and DHA intake of 500 mg/day or more [29].

^b^Adjusted for age, sex, ethnicity, energy intake, body mass index, mean arterial blood pressure, axial length, and fellow vessel caliber. Sex was not adjusted in the final model when analyzing boys and girls separately.

**Table 5:** Association between quartiles of long chain omega-3 polyunsaturated fatty acids (LCn-3 PUFA) and adjusted mean retinal vascular caliber in 17-year old girls.

	Retinal vessel caliber (μm), mean (95% CI) ^a^	
Dietary intake (mg/ day)	Arteriolar caliber	Venular caliber
Total LCn-3PUFA		
1^st^ quartile (≤116.2), n = 165	160.9 (159.2–162.5)	234.2 (231.4–237.0)
2^nd^ quartile (116.3–255.3), n = 166	161.6 (159.9–163.4)	235.1 (232.1–238.0)
3^rd^ quartile (255.5–438.1), n = 166	162.1 (160.6–163.7)	236.5 (233.9–239.2)
4^th^ quartile (≥443.1), n = 166	164.8 (163.2–166.4)	231.8 (229.0–234.7)
*p*-trend	0.001	0.10
EPA		
1^st^ quartile (≤34.7), n = 165	160.6 (158.9–162.2)	234.8 (232.0–237.7)
2^nd^ quartile (34.7–88.4), n = 166	161.9 (160.3–163.6)	234.8 (231.9–237.7)
3^rd^ quartile (88.4–166.2), n = 166	162.4 (160.9–164.0)	236.3 (233.5–239.1)
4^th^ quartile (≥166.9), n = 166	164.3 (162.8–165.9)	231.9 (229.2–234.7)
*p*-trend	0.0004	0.05
DPA		
1^st^ quartile (≤33.6), n = 165	160.5 (158.8–162.1)	236.0 (233.1–238.9)
2^nd^ quartile (33.8–53.5), n = 166	163.0 (161.3–164.6)	233.4 (230.6–236.3)
3^rd^ quartile (53.6–78.8), n = 166	162.9 (161.2–164.5)	234.0 (231.1–236.8)
4^th^ quartile (≥79.0), n = 166	163.6 (161.7–165.4)	234.0 (230.8–237.2)
*p*-trend	0.08	0.39
DHA		
1^st^ quartile (≤37.4), n = 165	161.2 (159.6–162.8)	234.2 (231.5–236.9)
2^nd^ quartile (37.7–116.7), n = 166	161.8 (160.0–163.6)	235.0 (232.0–238.0)
3^rd^ quartile (116.9–205.0), n = 166	162.0 (160.5–163.6)	236.5 (233.8–239.1)
4^th^ quartile (≥206.3), n = 166	164.5 (162.8–166.1)	231.9 (229.1–234.7)
*p*-trend	0.003	0.16

^a^Values are adjusted means; 95% CIs in parentheses. Values were calculated by using ANCOVA and were adjusted for age, ethnicity, energy intake, body mass index, mean arterial blood pressure, axial length, and fellow vessel caliber.

**Table 6:** Association between quartiles of long chain omega-3 polyunsaturated fatty acids (LCn-3 PUFA) and adjusted mean retinal vascular caliber in 17-year old boys.

	Retinal vessel caliber (μm), mean (95% CI) ^a^	
Dietary intake	Arteriolar caliber	Venular caliber
Total LCn-3PUFA		
1^st^ quartile (≤128.9), n = 134	159.3 (157.6–160.7)	229.9 (226.9–232.8)
2^nd^ quartile (128.9–231.2), n = 134	159.1 (157.7–161.5)	230.6 (227.9–233.3)
3^rd^ quartile (232.6–416.2), n = 134	158.9 (157.3–160.5)	232.3 (229.5–235.1)
4^th^ quartile (≥417.3), n = 134	158.6 (156.9–160.2)	231.7 (228.9–234.6)
*p*-trend	0.63	0.46
EPA		
1^st^ quartile (≤35.6), n = 134	158.8 (157.2–160.5)	229.9 (226.9–232.8)
2^nd^ quartile (36.1–74.1), n = 134	158.9 (157.4–160.3)	232.0 (229.3–234.7)
3^rd^ quartile (75.8–131.6), n = 134	159.7 (158.1–161.3)	231.1 (228.3–233.9)
4^th^ quartile (≥131.9), n = 134	158.5 (156.8–160.2)	231.6 (228.6–234.6)
*p*-trend	0.44	0.32
DPA		
1^st^ quartile (≤40.1), n = 134	159.0 (157.1–160.9)	232.2 (229.0–235.4)
2^nd^ quartile (40.4–58.3), n = 134	158.5 (156.9–160.0)	231.8 (229.0–234.6)
3^rd^ quartile (58.4–82.1), n = 134	159.7 (158.1–161.3)	228.5 (225.7–231.2)
4^th^ quartile (≥82.1), n = 134	158.8 (157.1–160.6)	232.1 (229.1–235.0)
*p*-trend	0.90	0.77
DHA		
1^st^ quartile (≤45.1), n = 134	159.0 (157.3–160.7)	229.8 (226.8–232.8)
2^nd^ quartile (45.2–98.1), n = 134	159.0 (157.6–160.4)	232.0 (229.4–234.7)
3^rd^ quartile (99.1–196.7), n = 134	158.7 (157.1–160.4)	230.9 (228.0–233.8)
4^th^ quartile (≥196.8), n = 134	159.2 (157.6–160.8)	231.6 (228.7–234.5)
*p*-trend	0.84	0.67

^a^Values are adjusted means; 95% CIs in parentheses. Values were calculated by using ANCOVA and were adjusted for age, ethnicity, energy intake, body mass index, mean arterial blood pressure, axial length, and fellow vessel caliber.

## Discussion

Findings from this community-based cohort show that mean intake of fish/seafood as well as total LCn-3 PUFA, EPA and DHA increased during adolescence. However, our findings suggest that mean consumption levels were low during childhood or adolescence. Ethnicity was a significant predictor of meeting national recommendations for fish, EPA and DHA intake. We provide novel epidemiological data showing that fish consumption and LCn-3 PUFA intake in adolescent girls is positively associated with mean retinal arteriolar caliber. These observed associations are in agreement with the published literature showing a potential for microvascular benefits from fish and diets rich in LCn-3 PUFA [15,22], even as early as adolescence.

Fish/ seafood consumption and LCn-3 PUFA intake in our study participants were higher than reported levels in previous studies of Australian children and adolescents [11,12]. The different dietary data collection methods and different nutrition composition databases which were used for analysis could explain these observed discrepancies in intake levels. Although dietary intakes observed in this cohort were generally higher, the mean intake levels were still low. Specifically, the suggested dietary targets for LCn-3 PUFA are 610 mg/d for males aged ≥14 years and 430 mg/d for females aged ≥14 years [37]; although in our study, 17-year old boys and girls consumed only 333.9 and 318.1 mg/day, respectively. This is substantially lower than the national recommended dietary targets. Further, just under half of schoolchildren were still not meeting recommended levels of fish consumption during adolescence [29]. However, it is important to recognize that there are currently insufficient data on what constitutes an optimal level of LCn-3 PUFA and fish intake in children. This is because dietary recommendations are generally for the 90th percentile level of intake in the population, and based on the recognition of a large body of evidence that higher levels of LC n-3 are consistently related, in observational studies, to better health and lower chronic disease risk. Nevertheless, these low levels of consumption are still of concern given that adolescence is a critical period during which lifetime habits are established [38,39]; and that inadequate LCn-3 PUFAs intake could have detrimental effects at this stage of human life including on neurological function and vascular health [1,2]. However, our findings need to be interpreted with caution as in children, FFQs are known to commonly over-report dietary intake, and in general are not able to capture energy intake reliably [40]. Although we had taken steps in the FFQ data cleaning process to exclude participants who were under or over-reporters [39].

Of the sociodemographic factors (e.g. parental education, employment status) that could potentially influence LCn-3 PUFA and fish intake in the longer term, only ethnicity was found to be a significant predictor. Most studies report high consumption of fish and seafood in East Asians [41]. The results of our study support those prior reports and showed that East Asians maintained their high intake during adolescence. Moreover, results of LCn-3 PUFA intake among East Asians were also consistent with their fish consumption levels during adolescence. These observed findings suggest that future research is needed that into how ethnicity could influences fish/ seafood consumption, which in turn could enhance the ability of intervention strategies to improve health and lifestyle outcomes for these adolescents from specific ethnic groups.

We demonstrate a novel, cross-sectional association between higher intakes of fish, LCn-3PUFA, EPA and DHA and wider retinal arterioles in adolescent girls. Widening of retinal arteriolar caliber is a beneficial structural change, which is associated with lower risk of CVD and cerebrovascular diseases [16,42,43]. These data are consistent with our prior adult study showing increasing consumption of fish to be associated with wider mean retinal arteriolar diameter [15]. As ours is an epidemiological study, we can only speculate on possible mechanisms by which fish and LCn-3PUFA independently influences retinal arterioles. First, biomarkers of oxidative stress are thought to be associated with retinal arteriolar caliber [44]. Therefore, LCn-3PUFAs—EPA and DHA, as found in oily fish and fish oils, could influence retinal arteriolar caliber via their ability to decrease the production of inflammatory mediators (e.g., cytokines and reactive oxygen species) and the expression of adhesion molecules [5,45]. Second, it has been shown that acetylcholine-stimulated relaxation of small arteries taken from hyper-cholesterolemic patients was significantly improved after three months of supplementation of EPA+DHA [46]. Fish oil intake has also been shown to improve endothelial function [47] and to increase arterial compliance [48]. These effects may be secondary to fish oil’s ability to enhance nitric oxide production [49,50], and may be another pathway by which fish and LCn-3PUFA consumption beneficially influences retinal arteriolar structure. Although, in our study we only had information about EPA+DHA from diet, and supplement use was not collected in this study. Further, we caution that this could be a chance finding and requires confirmation by other prospective adolescent studies.

It is unclear why associations with retinal arteriolar caliber were observed primarily in girls and not boys. One possibility is that as boys and girls reach puberty, sex hormones could mediate the differential effects of dietary factors such as LCn-3PUFA and fish on the retinal arterioles. This is consistent with our prior childhood study which showed carbohydrate nutrition variables were associated with retinal microvascular signs in girls but not boys [36]. Further, it supports published research showing that women have greater susceptibility to developing small vessel disease than men, including coronary artery disease [51,52]. We have also previously shown in a meta-analysis that narrower retinal arterioles are more strongly associated with incident coronary heart disease in women than in men [16].

These data concur with our previous studies showing that diet and early lifestyle choices are associated with microcirculatory health, which is likely related to the risk of CVD in later life [36,53]. While the observed differences in retinal arteriolar caliber across the range of LCn-3PUFA intake was relatively modest (~2–3% difference) in girls, these small reductions in retinal vessel diameter could still have some clinical relevance. This because even a 1.1-μm reduction in retinal arteriolar caliber was previously shown to be associated with a 10-mm Hg increase in systolic BP [54,55]. Moreover, given the consistency of published findings between structural changes in retinal arterioles and CVD events, risk factors and pathology [19–21,56], it is reasonable to infer that any measurable change to the retinal microvasculature in adolescents (i.e. subtle retinal arteriolar widening) could be a subclinical marker signaling reduced risk of CVD, particularly hypertension, in later life [57,58]. Therefore, these epidemiological data provide further support for the development of targeted public health strategies such as nutrition education and behavior modification strategies to increase the consumption of fish and seafood, and LCn-3PUFA supplementation or incorporation of foods that are enriched with LCn-3PUFA (e.g. certain brands of bread, milk and eggs)[59] among children and adolescents.

Strengths of this study are it is a representative sample of children/adolescents, prospective design, and the use of a validated long FFQ tool to determine dietary intakes [24]. However, there are some noteworthy study limitations. First, we are unable to prospectively analyze retinal vessel structure given that differing cameras were used at the two examinations. Also, we had not collected data on pubertal signs and this is a potential study limitation, as we cannot disregard the possibility of a confounding influence from puberty [53]. We also cannot disregard the potential for residual confounding from factors that were either not measured or unable to be collected in our study e.g. parental dietary intakes, inflammatory markers and red blood cell fatty acids (valid biomarker of habitual LCn-3PUFA). Moreover, there could have been residual confounding from ethnicity on observed associations, this is because ethnicity of the child was determined only if both parents shared that ethnic origin. The inclusion of children with e.g. mixed Asian and Caucasian ethnicity could have influenced genetic factors that influence vessel caliber and fish intake, which we are not able to account for in this study. Finally, FFQs are known to commonly over-report or under-report dietary intake, and in general are not able to capture energy intake reliably [40]. Nevertheless, we had taken steps in the FFQ data cleaning process to exclude participants who were under or over-reporters [60]. Further, the FFQ used in our study was not specifically validated for LCn-3 PUFA intakes, hence, we can only use diary fat intake as a proxy (for which the correlation coefficients indicate reasonable estimates) [24]. However, intake of LCn-3 PUFA is primarily based on a limited range of foods [61], mostly fish and seafood; and given that the FFQ included a reasonable set of questions on these items (separate items on crumbed, fresh, canned and shellfish), it is likely to provide a reasonable estimate of LCn-3 PUFA intake in our cohort.

In summary, this community-based study showed that over 5 years there was an appreciable increase in the dietary intakes of LCn-3 PUFA, EPA and DHA among Australian schoolchildren. However, this study also highlighted the issue of children and adolescents not consuming the recommended amounts of LCn-3PUFA, which is likely due to the low fish consumption observed in this cohort. We provide novel epidemiological evidence suggesting that diets rich in LCn-3 PUFA and fish are associated with a healthy retinal microvasculature profile during adolescence and potentially, provides further support for increasing the habitual consumption of fish and LCn-3PUFA as part of targeted CVD prevention programs for adolescents.

## Supporting information

S1 Table Baseline characteristics of study participants versus non-participants. (DOC)
