# Supplementary material for: Pattern of omega-3 polyunsaturated fatty acid intake and fish consumption and retinal vascular caliber in children and adolescents: A cohort study
Source: PLoS One. 2017 Feb 13;12(2):e0172109. doi: 10.1371/journal.pone.0172109 (PMC5305252; doi:10.1371/journal.pone.0172109)
Supplement: S1 Table — (DOC) [file pone.0172109.s001.doc]

**Online Supplementary Table 1.** Baseline characteristics of study participants versus non-participants

| Characteristics | Participants (n=633) | Non-participants (n=1720) | P-value |
| --- | --- | --- | --- |
| Age | 12.74 (0.4) | 12.69 (0.5) | 0.01 |
| Male | 282 (44.6) | 908 (52.8) | 0.0004 |
| Ethnicity, % |  |  |  |
| Caucasian | 413 (65.2) | 993 (57.7) | 0.001 |
| East Asian | 104 (16.4) | 248 (14.4) | 0.23 |
| South Asian | 31 (4.9) | 98 (5.7) | 0.45 |
| Middle Eastern | 34 (5.4) | 132 (7.7) | 0.05 |
| Other | 51 (8.1) | 249 (14.5) | <0.0001 |
| Parental education^a^ | 286 (48.5) | 553 (37.6) | <0.0001 |
| Parental employment ^b^ | 547 (93.3) | 1330 (88.8) | 0.002 |
| BMI | 20.07 (4.2) | 20.53 (4.2) | 0.02 |
| MABP | 81.16 (8.1) | 80.85 (8.5) | 0.43 |
| Axial length | 23.35 (0.9) | 23.40 (0.8) | 0.21 |

Data is presented as mean (SD) or n (%)

^a^ Parents who had attained tertiary level education (greater than high school).

^b^ Parents who were in full or part-time employment.
